# Nanofabrication: the unsung hero in enabling advances in nanophotonics

**DOI:** 10.1515/nanoph-2023-0217

**Published:** 2023-04-20

**Authors:** Pan Chengfeng, Zhang Shutao, Maria Farsari, Sang Hyun Oh, Joel K. W. Yang

**Affiliations:** Engineering Product Development, Singapore University of Technology and Design, 487372 Singapore, Singapore; FORTH/IESLN, Plastira 100, 70013, Heraklion, Crete, Greece

Nanophotonics plays an important role in driving innovation in a growing number of fields over the last several decades. Many of the innovations that impact our daily lives, e.g. microscopy, biosensors, miniaturized spectrometers, imaging, and displays, are rooted in nanophotonics. Behind the scenes of the rapid development in nanophotonics is the ever-advancing nanofabrication technology. Nanofabrication is the vehicle that enables the realization of novel ideas in nanophotonics. However, despite the significant effort that goes into developing fabrication processes, techniques and hacks, they often get tucked away in appendices of thesis or supporting information of journal articles.

In this special issue, we celebrate nanofabrication by bringing it into the limelight. Through the compilation of 23 articles, we take a snapshot of some of the ingenious fabrication approaches in producing devices, enabling experiments, and demonstrating new nanophotonic concepts. The maturation of some nanofabrication technologies through turnkey systems, e.g. electron-beam lithography (EBL) offers reliable and unlimited possibilities for the design of photonic systems. Nonetheless, the integration and processing of new materials, e.g. nanoparticles, molecules and 2D materials, into nanophotonic structures offer opportunities for creative nanofabrication strategies. Here, we have grouped the articles in this special issue broadly into different fabrication processes.

## 1 Top-down lithography

EBL is utilized to fabricate high resolution nanostructures with the support of material addition or removal techniques. Thus, versatile photonic applications are demonstrated. im Sande et al. [1] fabricated single-celled metasurfaces to compare broadband and spin-multiplexed holograms with four different multiplexing strategies. Jung et al. [2] designed and fabricated metalenses to realize three-dimensional focal spot relocation. Zhang et al. [3] and de Jong et al. [4] present electrically tunable devices. Meunier et al. [5] demonstrate the first embedment of room-temperature telecom single-photon emitters. By integrating photonic device with semiconductor platform, Choi et al. [6] proposed an approach to fabricate photonic crystal structures that generate solitary waves on CMOS Chips. Oh et al. [7] reported the guided domino lithography (GDL) by controlling geometries and exposing dose to realize uniform ultra-sharp nanoantenna arrays. Wen et al. [8] review how significant beam divergence and polarization instability have been solved by integrating metasurfaces into semiconductor lasers.

Employing focused ion beam (FIB) lithography, Liu et al. [9], fabricated on-chip nano-cilia metasurfaces that operate in near-infrared and visible wavelengths. Wang et al. [10] applied direct laser interference patterning (DLIP) to direct fabricate the designed 2D quantum dot or quantum ring pattern. Using photolithography, Kim et al. [11] report a wafer-scale prepatterned crack process with an unprecedented mixture of macroscopic length and Angstrom-scale controllability. Logotheti et al. [12] demonstrates that the laser-induced forward transfer (LIFT) method can achieve a one-step, nondestructive printing of the prototypical 2D material MoS2. In addition, Bai et al. [13] investigated the morphologies of LIPSS on ZnO substrates by mask-less ultrafast laser processing. Paz-Buclatin et al. [14] developed a 3D laser nanolithography technique that enables the fabrication of mm-long hollow nanopores inside solid-state laser crystals.

## 2 Bottom-up lithography

Multi-photon polymerization (MPP) lithography is developed to realize more complicated nanostructures. Zhang et al. [15] combine it with up-conversion materials to realize tunable feature-size printing, while Samsonas et al. [16] studied 3D nanopolymerization and damage threshold dependence on laser wavelength and pulse duration, and Waller et al. [17] 3D printed metallic structures via direct laser writing. Ottomaniello et al. [18] fabricated conformable terahertz free-standing plasmonic absorbers. In addition, Somers et al. [19] studied the photoinhibition lithography with common photoinitiators to realize the high-throughput nanoprinting. Jiang et al. [20] demonstrate a metasurface broadband perfect absorber via the self-assembly based colloidal lithography.

## 3 Material removal

Olsson et al. [21] utilize PVD and colloidal lithography to integrate the electrochromic materials with the nanostructures which realize the electrical tuning of the colors. Kaufmann et al. [22] present a workflow to identify the parameter set offering the best etching results independent of the plasma system being used for Lithium niobate on insulator.

## 4 Material addition

Chia et al. [23] reviewed and discussed recent development in deuterated SiNx platforms with the help of low temperature, back-end-of-line CMOS-compatible fabrication plasma-enhanced chemical vapour deposition (CVD) process. Last but not least, Zhang et al. [24]. proposed a universal metasurface transfer technique for heterogeneous integration.

In conclusion, this special issue offers current research articles and review articles covering plentiful and advanced fabrication methods at different scale levels, resolution, throughput, and platforms. We hope this issue on nanofabrication can inspire renewed interest among students and researchers to link good design with novel and creative nanofabrication processes. We sincerely appreciate all contributions from the authors to this special issue and hope you enjoy reading this special issue as much as we did.
